# *Helicobacter pylori* Infection in Geriatric Patients: Current Situation and Treatment Regimens

**DOI:** 10.3389/fmed.2021.713908

**Published:** 2021-09-30

**Authors:** Qiuyue Huang, Xiaofen Jia, Yingming Chu, Xuezhi Zhang, Hui Ye

**Affiliations:** Department of Integrated Traditional Chinese and Western Medicine, Peking University First Hospital, Beijing, China

**Keywords:** *Helicobacter pylori*, eradication therapy, stomach cancer, dual therapy, complementary therapy, elderly

## Abstract

*Helicobacter pylori* (*H. pylori*) has so far infected more than half the global population. It is the most important and controllable risk factor for gastric cancer. The elderly, who are at a higher incidence of the infection, are also commonly found to develop antibiotic resistance. The symptoms, diagnosis, clinical features (of gastric or extra-digestive diseases), and treatment of *H. pylori* infection in the elderly, are different from that in the non-elderly. Health conditions, including comorbidities and combined medication have limited the use of regular therapies in elderly patients. However, they can still benefit from eradication therapy, thus preventing gastric mucosal lesions and gastric cancer. In addition, new approaches, such as dual therapy and complementary therapy, have the potential to treat older patients with *H. pylori* infection.

## Introduction

Aging is an inexorable process in the human life cycle. According to the World Health Organization, population aging is an evident phenomenon in all developed countries and some developing countries (1). Poor health of the elderly and the increasing disease burden have led to a great demand for medical care and social services (2, 3). Aging widely affects the functioning of various organs. Disrupted functioning of the digestive organs can lead to indigestion symptoms. A survey in Italy involving over 3,000 outpatients aged ≥60 years showed that over 40% of them had experienced gastrointestinal symptoms (4). Aging-related changes in the upper digestive system is mainly reflected in anatomical and physiological changes, such as gastric mucosa atrophy, decreased motility of the esophagus and stomach, reduced secretion of gastric acid and bile, decreased mucosal blood flow, and reduced digestive enzyme activity. Moreover, depending on factors such as infections, comorbidities, nutrition, and medications [e.g., non-steroidal anti-inflammatory drugs (NSAIDs)], aging can cause digestive disorders or further progression of diseases acquired in youth.

**Figure 1:**
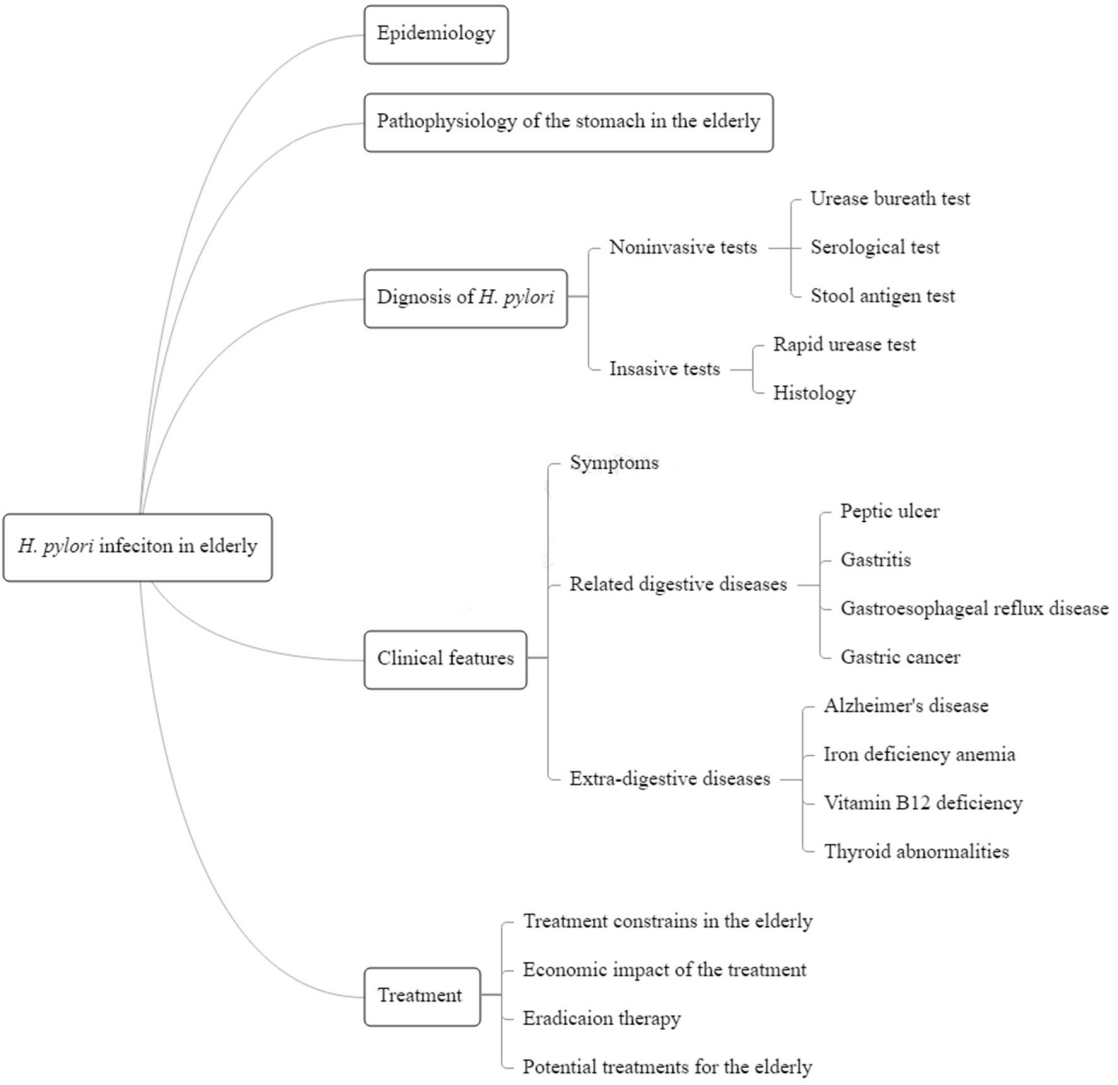
Summary figure of the review.

Among aged people, *Helicobacter pylori* (*H. pylori*), the most common infectious pathogen in the stomach, is the main risk factor for gastric cancer (5). Although *H. pylori* affects individuals of different age groups, the elderly have a higher prevalence of *H. pylori* infection, and risk of developing atrophic gastritis, and stomach cancer (6–8). As per a previous study, the effect of *H. pylori* eradication therapy in decreasing gastric cancer deaths varied among different age groups (failing in patients aged ≥80 years), but most of the aged patients with *H. pylori* infection could still benefit from the treatment (9). The eradication of *H. pylori* in elderly patients could be tricky due to decline in bodily functions, complications of other disease like renal failure, drug combinations, etc. (10). The use of mainstream therapies have to face more competing situations in older patients. The Fifth Chinese National Consensus Report on *H. pylori* infection has focused on geriatric patients, but it lacks information and treatment strategies specific to such patients (11). There is substantial scope to improve health care for elderly patients with *H. pylori*-related diseases. Therefore, this article aims to review the current knowledge on *H. pylori* infection in the geriatric population and the promising new approaches in this regard (Figure 1).

## Epidemiology

Approximately 50% of the global population is infected with *H. pylori*. The infection rate varies with age, socioeconomic class, and geographic area. *H. pylori* infection is usually acquired during childhood, and chronic infection lasts a lifetime if untreated. Due to improvement in sanitary conditions, the prevalence of *H. pylori* infection has gradually decreased in recent years, especially among the younger generation in the developed countries (12). However, in poor and developing countries and regions, the prevalence *of H. pylori* infection remains high in children. Decades later, these children might get affected by *H. pylori*-related diseases when they age (13, 14). The total *H. pylori* infection rate among the elderly in Beijing, China, according to a seroepidemiological investigation 15 years ago, was 83.4% (15), while in a recent study using the ^13^C-urea breath test, it was found to be 46.5% (16). Our previous study retrospectively collected the results of the ^13^C-urea breath test performed in our department from 2013 to 2019, in which the prevalence of *H. pylori* infection in people aged <20 years was 22.0%, and in those aged >60 years, it was 39.4% (17). The geriatric population in developing countries such as China is still facing the challenge of a high incidence of *H. pylori* infection. In addition, antibiotic resistance is more severe in the elderly population. A study conducted in China revealed that the antibiotic resistance rates to clarithromycin, levofloxacin, and metronidazole were 17.76, 19.66, and 95.5%, respectively in the entire patients; the highest antibiotic resistance rate was observed in patients aged 71–80 years (18). A larger study found that younger patients exhibited lower antibiotic resistance rates compared to patients aged ≥40 years (19). Thus, the epidemiology of *H. pylori* infection in elderly patients is characterized by high prevalence and drug resistance.

## Characteristics of Aging Stomach

Compared with normal stomach, the stomach of the elderly exhibits functional and structural abnormalities, which lead to a series of biological responses, including impaired nuclear-mitochondrial communication (20), hypoxia (21), apoptosis (22), and increased levels of reactive oxygen species. The common characteristics of aging stomachs include slowing of gastric emptying, and dyspepsia (23). It also results in ‘anorexia of aging' (24) and post-prandial hypotension (25). Further, aging also contributed to a decrease in microbial diversity in stomach. In *H. pylori*-uninfected gastric corpus mucosa, the abundance and the microbial diversity of Firmicutes (*Streptococcus*, and *Veillonella*), Fusobacteria (*Fusobacterium*), Actinobacteria (*Rothia*), and Bacteroidetes (*Prevotella*) both decreased with age (26). In addition, atrophy of the gastric glands and the decrease in gastric mucosal blood flow weaken the gastric mucosal defense. The atrophy of the gastric glands induces the reduction of bicarbonate and prostaglandin production in the stomach and impairs the mucosal barrier. Gastric blood vessels are important for facilitating blood flow and preserving gastric mucosal morphology (27). They maintain the mucosal barrier, provide nutrients and oxygen, and defend the gastric mucosa against injury (28). As reported in a study, in elderly people, the mucosal blood flow decreases by >60%, causing profound hypoxia in all mucosal cells (29). The fundamental process of angiogenesis is impaired in aging gastric mucosa (30). These changes lead to increased susceptibility to injuries, such as the NSAID. *H. pylori* infection induces overproduction of nitric oxide, upregulates endothelial nitric oxide synthase expression, and induces angiogenesis (31), thus leading to obstruction of oxygen and blood flow.

*H. pylori* infection causes stomach mucosal lesions, atrophy, and microcirculation dysfunction in older people. Eradication therapy for *H. pylori* infection in geriatric patients can potentially prevent gastric cancer (32). The testing for *H. pylori* should be performed in aspirin and NSAID users (33). *H. pylori* eradication can prevent gastric mucosal injury induced by antiplatelet drugs (34). However, due to the complicated comorbidities, history of medications, and poor adherence to eradication therapy among the older patients, further efforts are required to develop appropriate treatment strategies for them.

## Diagnosis

### Non-invasive Tests

Non-invasive tests include the urea breath test (UBT), *H. pylori* stool antigen test, and serological tests (35, 36). UBT is considered the gold standard of non-invasive methods for *H. pylori* diagnosis (37). It is the first choice for treating outpatients because of its wide availability and high accuracy (96%). In a previous study, the mean UBT values were found to significantly increase with age (38). The manufacturer-recommended cut-off values for UBT are not applicable in every setting. The ^13^C-UBT might have higher threshold values in subjects aged >60 years due to impairment in gastric emptying or lower endogenous CO_2_ production, compared to younger people (39). Severe gastric mucosal atrophy and intestinal metaplasia are independent risk factors for false positivity of UBT (40) because of the resultant lowered *H. pylori* density in the stomach (41). Thus, the UBT cut-off values should take age of the patient into account (42).

As per a study, the sensitivity, specificity, and diagnostic accuracy of serological tests in elderly patients were 74.4, 59, and 67%, respectively (43). The combined use of serological tests with UBT may further increase the sensitivity and specificity of *H. pylori* detection. However, the *H. pylori* antibody test may often yield false negative results because of spontaneous disappearance of *H. pylori* with advanced gastric mucosal atrophy, particularly in individuals aged ≥65 years (44).

*H. pylori* stool antigen test can effectively compensate for the drawbacks of UBT in the elderly. Detection of bacterial antigens was not affected by atrophic gastritis, ulceration, or intestinal metaplasia. In a study that evaluated the diagnostic efficiency of *H. pylori* stool antigen test in elderly subjects (≥65 years old), the accuracy (91.5%) and specificity (97.6%) were both high for all participants. However, the sensitivity was only 68.7%. Further, it was found that constipation and colorectal polyps were negatively and positively associated with its sensitivity, respectively (45).

### Invasive Tests

Rapid urease test (RUT) is the preferred method for invasive detection of *H. pylori*, with a sensitivity of up to 95% and specificity of 85–95% (46). The sensitivity of RUT performed on antral biopsies of patients aged ≥60 years (57%) was lower than that performed on younger patients (75%) (47). Histological diagnosis of *H. pylori* can be performed at the same time as the pathological diagnosis in the gastric mucosa. For patients with high risk of gastric cancer, histological detection is recommended, which is of great significance for the evaluation of prognosis and the formulation of treatment measures. Upper gastrointestinal endoscopy is often necessary for the diagnosis of *H. pylori* infection in older adults with varying abdominal symptoms because of the high prevalence of severe stomach disorders in this age group (48). Bacterial culture performed on gastric biopsy specimens is mainly used for scientific research and is not a routine method for *H. pylori* detection. It showed low sensitivity (53.3%) and high specificity (100%) (49).

## Clinical Presentation

### Symptoms

*H. pylori* infection is associated with the occurrence of peptic ulcers, chronic atrophic gastritis, gastric cancer etc. (50). With increasing age, the incidence and severity of most upper gastrointestinal diseases increase. The clinical features of *H. pylori* infection range from asymptomatic gastritis to gastrointestinal malignancy (51). Symptoms such as nausea, vomiting, epigastric pain, and belching are also common in acute *H. pylori* infection. However, most symptoms improve or disappear gradually in persistent chronic infection. In case of old patients, long-term *H. pylori* infection usually has no or mild digestive symptoms. These non-specific symptoms are often confused with those of other diseases, especially in case of *H. pylori*-induced gastritis. Patients with treatment-resistant *H. pylori* infections show more severe gastrointestinal symptoms (52). Further, older people with a history of treatment failure and excessive antibiotic exposure might also have more prominent symptoms.

### Peptic Ulcer Disease

*H. pylori* infection is strongly associated with peptic ulcer disease (PUD) (53). As the population ages, the incidence of cardiovascular and musculoskeletal diseases increases, as does the use of aspirin and NSAIDs. Aspirin, NSAIDs, and *H. pylori* are independent risk factors for PUD and its complications (54–56). The incidence of PUD increases with age, with most ulcers occurring between the ages of 25 and 64 (57). The most common and serious complication of PUD is bleeding, and people aged >60 years are at the highest risk of suffering from it (58). Treatment for *H. pylori*-associated PUD is mainly directed toward the eradication of infection. In cases of NSAID-induced ulcers, *H. pylori* eradication alone did not impair ulcer healing; and the use of proton pump inhibitors (PPI), H_2_ receptor antagonists, and misoprostol are recommended before using NSAIDs, in order to protect the gastric mucosa and prevent PUD and its complications (33, 55, 58). Early eradication of *H. pylori* in patients with PUD can reduce the risk of gastric cancer (59).

### Chronic Atrophic Gastritis

Chronic atrophic gastritis (CAG) is a disease characterized by atrophy of the gastric glands and/or intestinal metaplasia, which is mainly caused by prolonged persistence of *H. pylori* infection. Studies on the prevalence of CAG in different parts of the world have found that CAG is relatively common among the elderly (7). A study from Germany found that the prevalence of chronic atrophic gastritis increased from 4.8% in the 50–54 age group to 8.7% in the 70–74 age group (6). CAG is also a precancerous lesion of gastric cancer, especially when accompanied by intestinal metaplasia. All *H. pylori-*positive individuals should receive eradication therapy unless there are competing considerations (60). The eradication of *H. pylori* can reverse the atrophy of the gastric antrum and gastric body to a certain extent, but it has no observable effect on intestinal metaplasia (61). In the elderly, however, eradication of *H. pylori* may improve gastric atrophy and prevent the progression of intestinal metaplasia (8). Further, it can also delay the progression of CAG in the elderly, which is of great significance for the prevention of gastric cancer (62).

### Gastroesophageal Reflux Disease

Gastroesophageal reflux disease (GERD) is a multifactorial disease that causes reflux of acidic stomach contents into the esophagus, causing tissue damage. Under normal conditions, gastric acid secretion does not decrease with age, but factors that cause atrophic gastritis, such as *H. pylori* infection, reduce gastric acid secretion (36). A case-control study found that compared with young and middle-aged GERD patients, atypical symptoms, low upper esophageal sphincter resting pressure, increased incidence of ineffective esophageal motility, and acid exposure were more significant in the elderly (63). A meta-analysis indicated that the prevalence of GERD was significantly higher in patients with *H. pylori* eradication than in those without it (64). However, eradication of *H. pylori* may not affect the healing rates or relapse rates of preexisting GERD (65). For patients with GERD, their condition should be comprehensively evaluated before carefully initiating *H. pylori* eradication therapy.

### Gastric Cancer

Gastric cancer is the fifth most common cancer and the third most common cause of cancer-related deaths worldwide (66). More than 1 million new cases of gastric cancer were reported in 2018, with an estimated 783,000 deaths (equivalent to 1 in 12 deaths worldwide). East Asian countries have a high incidence of gastric cancer (67). *H. pylori* is the main risk factor for gastric cancer apart from diet, alcohol, and smoking (67). *H. pylori* eradication may reduce the risk of gastric cancer in healthy, asymptomatic, and gastrointestinal patients (68). Among the high-incidence- and high-risk-population of gastric cancer, screening and eradication of *H. pylori* are recommended before the development of atrophic gastritis and intestinal metaplasia to prevent gastric cancer (14, 69). Due to the high incidence of gastric mucosal atrophy and intestinal epithelial metaplasia in elderly patients, it was thought in the past that the benefit of *H. pylori* eradication might be limited to treating gastric cancer. However, a long-term cohort study demonstrated that the risk of gastric cancer was significantly lower in people aged ≥60 years than that in the general population aged ≥10 years, after eradication of *H. pylori* (32). Moreover, patients at early stages of gastric cancer who received *H. pylori* eradication therapy had lower rates of metachronous gastric cancer than those who did not (70).

### Extra-Digestive Diseases

Although the relationship between *H. pylori* infection and gastric pathology has been established, the effect of this bacterium on the host body as a whole remains to be studied. It is well-known that some pathogens exist locally and may cause systemic pathological effects (71). Studies have found that *H. pylori* is associated with blood disorders such as vitamin B12 deficiency, iron-deficiency anemia (IDA), primary immune thrombocytopenia, as well as a number of dermatological, eye, metabolic, neurological, and allergic diseases (71). Among these diseases, we will only elaborate on diseases that have a greater impact on the elderly.

Approximately 46.8 million people live with dementia worldwide in 2015 (72). Alzheimer's disease (AD) is the single biggest cause of dementia, and is doubling in prevalence every 5 years after age 65 (73). Epidemiological studies have shown an association between *H. pylori* infection, mild cognitive impairment, and AD (74). *H. pylori* may affect the pathophysiology of AD in various ways (74, 75). Selective eradication of potentially curative *H. pylori* infection is recommended for *H. pylori*-infected patients with AD, and those who might fulfill the criteria of clinicians (for example, poor response to conventional drugs and complicated gastrointestinal symptoms) (74).

Iron deficiency is a common cause of anemia in the elderly. Many studies have shown a strong link between *H. pylori* infection and IDA. Compared with uninfected individuals, the prevalence of IDA was higher, and iron levels were significantly lower in those infected with *H. pylori* (76, 77). Hemorrhage (i.e., bleeding from ulcers) resulting from blood loss and the reduced iron absorption are the main causes of IDA (71). The Maastricht V/Florence Consensus Report recommends that patients with IDA of unknown cause should be diagnosed and treated for *H. pylori* (33). *H. pylori* eradication therapy combined with iron supplementation may help improve ferritin and hemoglobin levels (76).

Vitamin B12 deficiency is common in the elderly, and its prevalence increases with age (78, 79). Chronic atrophic gastritis, ingestion of drugs that affect gastric acid secretion or acid (i.e., proton pump inhibitors and antacids), *H. pylori* infection, overgrowth of intestinal bacteria, inadequate food intake, alcohol abuse, and smoking can all lead to B12 deficiency (79, 80). The clinical manifestations of B12 deficiency are heterogeneous; generally, there are no visible clinical symptoms. Its classical manifestations include Hunter's glossitis, megaloblastic anemia, and subacute combined degeneration of the spinal cord, and are associated with atherosclerotic vascular disease and neuropsychiatric disorders (78). B12 deficiency is particularly difficult to diagnose in elderly individuals. The Maastricht V/Florence Consensus Report recommends that all patients with vitamin B12 deficiency be screened for *H. pylori* infection, and treated if necessary (33).

Persistent active inflammation induced by *H. pylori* infection can lead to autoimmune immunopathological reactivity in affected patients, including thyroid abnormalities. The prevalence of anti-thyroid peroxidase antibody positivity is more frequent in subjects with *H. pylori* infection, and the association was still significant after adjusting other confounding factors (81). *H. pylori*-mediated chronic inflammation has also been shown to increase the expression of proinflammatory cytokines. And non-thyroidal-illness syndrome may develop on this background of chronic inflammation. A positive association between chronic active *H. pylori* infection and non-thyroidal-illness syndrome prevalence in elderly male cohort was found (82).

## Treatment

Several clinical guidelines are available for treating *H. pylori* infection in adults (11, 33, 83–86), children, and adolescents (87, 88). In general, the regimens recommended for adults are effective for older patients. However, a comprehensive benefit/risk assessment should be performed and individualized treatment be given to eradicate *H. pylori* in elderly patients (11, 34), due to their declining physical conditions, serious comorbidities, side effects and ongoing medications (89). A retrospective study indicated that clinicians might be reluctant to treat very old patients, possibly due to concerns about complications (90). Age, physical health condition, and history of medications are the determining factors for performing *H. pylori* eradication therapy.

### Constraints in Treatment

The elderly population is more likely to be resistant to antibiotics because of excessive use in the past (91, 92). A multicenter cohort study showed that people aged >40 years were particularly at risk of developing dual drug resistance to levofloxacin and metronidazole (93). In case of chronic infectious bronchial pneumonia in the elderly, it is important to be wary of levofloxacin resistance, as they may have already been treated with quinolones or fluoroquinolones (94). It is recommended to select antibiotics according to the results of antibiotic susceptibility test. If eradication treatment fails, multidrug resistance may develop, making antibiotic selection more difficult (95, 96).

Aging stomachs are less defensive and more sensitive to stimuli, and the side effects of medications may be more pronounced, which may lead to less compliance to the regimen. Progressive aging of the human body can also change the ecological flora. The core microflora, such as Bacteroidetes, decreases in number, while the colony abundance of subdominant microflora such as Firmicutes, Actinobacteria, and Proteobacteria increases (97). The intestinal flora of the elderly population is more likely to be affected by antibiotic treatment as compared to that of the general population. Using probiotics during or after eradicating *H. pylori* may prevent or reduce microecological imbalance in elderly patients, thus reducing the occurrence of adverse effects (98, 99).

In addition to physiological senility, elderly people often suffer from a variety of diseases, such as hypertension, diabetes, liver and kidney dysfunction, etc. These diseases limit the use of certain drugs. For instance, the doses of amoxicillin and clarithromycin may have to be adjusted in patients with renal insufficiency or severe liver impairment. Further, comorbidities in the elderly patients result in drug interactions that can cause serious side effects (100). Some PPIs such as omeprazole can easily interact with cardiovascular drugs such as clopidogrel, which are commonly used in the geriatric population (101, 102). Amoxicillin, clarithromycin, metronidazole, and tetracycline may also interact with cardiovascular medications, such as statins, antiarrhythmic drugs, and warfarin (101).

### Economic Impact

Some economic analyses suggest that *H. pylori* screening and eradication are cost-effective for preventing gastric cancer, especially in high-risk areas (103–111). A study in Japan found that from 2013 to 2019, *H. pylori* eradication was economical and reduced the incidence of and mortality caused by gastric cancer in patients aged 20–80 years compared to those not having undergone eradication. Among the patients that had received eradication therapy, those aged 60 years had the highest cost benefits and best health outcomes (110). The use of *H. pylori* eradication as a strategy for the prevention of gastric cancer can not only save lives, but also greatly reduce the healthcare-related economic burden on the national budget (110).

### Eradication Regimens

Triple therapy has been used worldwide for decades and is the standard first-line eradication regimen for *H. pylori* infection (112). However, increased antibiotic resistance, particularly to clarithromycin, metronidazole, and levofloxacin (113, 114), has reduced the eradication rate of triple therapy (115, 116). A case-control study assessed the efficacy of a standard *H. pylori* eradication therapy among elderly patients, and concluded that the elderly did not affect efficacy or safety of a clarithromycin-based triple therapy for *H. pylori* eradication (117). Triple therapy was only recommended in the areas with clarithromycin resistance rate of <15%. Bismuth-containing quadruple therapy, concomitant therapy, sequential therapy, hybrid therapy, levofloxacin triple therapy, and rifabutin triple therapy were developed to overcome the challenge of antibiotic resistance (Table 1). These regimens are recommended by different consensus and guidelines for certain cases of *H. pylori* infection (33, 83, 84). Few studies have evaluated the efficacy and safety of these therapies in elderly patients. Bismuth-containing quadruple therapy was successful for the initial eradication of *H. pylori* in elderly patients, but 28% of patients had mild to moderate side effects (118). The sequential treatment regimen achieved significantly higher eradication rates in comparison with standard triple therapy in elderly patients, and there were mild side effects (<12%) of both the regimens (119). A retrospective investigation of 1,271 cases of *H. pylori* infection in elderly patients indicated that the rate of side effects with both the first and second treatment using triple therapy was <10%, suggesting that clinicians need not withhold treatment strictly based on old age (90).

**Table 1:** Current mainstream regimens.

**First line/rescue therapy**	**Eradication regimens**	**Definition**	**Duration**
Restricted option^a^/–	Classic triple therapy	PPI+AMO+CLA	14 d
		PPI+AMO+MTZ	
		PPI+CLA+MTZ	
Recommended/recommended	Bismuth quadruple therapy	PPI+B+TET+MTZ	10–14 d
Recommended/undetermined	Concomitant (non-bismuth quadruple) therapy	PPI+AMO+CLA+MTZ	10–14 d
Not recommended/not recommended	Sequential (non-bismuth quadruple) therapy	PPI+AMO followed by PPI+CLA+MTZ	5–7 d 5–7 d
Conditional recommended by ACG/–	Hybrid therapy	PPI+AMO followed by PPI+CLA+MTZ	7 d
Not recommended/recommended	Levofloxacin triple therapy	PPI+AMO+LFX	10–14 d
Conditional recommended by ACG/–	Levofloxacin sequential therapy	PPI+AMO followed by PPI+AMO+LFX+MTZ	5–7 d 5–7 d
Conditional recommended by ACG/–	LOAD therapy	PPI+LFX+MTZ+ doxycycline	
–/restricted option^b^	Rifabutin triple therapy	PPI+AMO+RFB	10 d
–/Recommended	Modified (high-dose) dual therapy	PPI+AMO	14 d

*The table is developed according to the Toronto Consensus guidelines, Maastricht V /Florence Consensus and American College of Gastroenterology Clinical Guideline*.

a *Restricted to areas with low clarithromycin resistance (<15%)*.

b *Restricted to cases in whom at least 3 recommended options have failed*.

*ACG, American College of Gastroenterology Clinical Guideline; AMO, amoxicillin; B, bismuth; CLA, clarithromycin; LFX, levofloxacin; LOAD, levofloxacin, omeprazole, alinia and doxycycline; MTZ, metronidazole; PPI, proton pump inhibitor; RFB, rifabutin*.

### Potential Treatments

High-dose dual therapy consisting of amoxicillin and a PPI has drawn much attention recently because of the low resistance to amoxicillin and simpler drug composition as compared to the quadruple therapies. It has been recommended by the ACG Clinical Guidelines as a salvage regimen (84). A systematic review and meta-analysis indicated that the efficacy and compliance of modified dual therapy was comparable with the current mainstream first-line regimens for *H. pylori* infection, with a significantly lower incidence of adverse side effects (120, 121). A retrospective, real-life study demonstrated that modified dual therapy consisting of high-dose amoxicillin and rabeprazole was effective and safe for the first-line treatment of *H. pylori* infection in elderly patients (122). Although the dosage and frequency are increased in modified dual therapy, the total dose throughout the day remains within the safe range. Dual therapy reduces the use of antibiotics and bismuth, which is a promising new approach for the elderly. However, it must be used in patients not allergic to penicillin and those not having renal insufficiency. A randomized controlled clinical trial showed that the cost of medications in the modified dual therapy was lower than that in bismuth-containing quadruple therapy (123).

The development of new drugs has also widened the options for eradication therapy in the elderly population. Vonoprazan, a novel potassium-competitive acid blocker, is more potent and long-acting than traditional PPIs (124, 125). According to a network meta-analysis, vonoprazan-based triple therapy achieved high eradication rates of more than 90% as a first-line empiric treatment. Vonoprazan-based triple therapy is highly effective and well-tolerated, regardless of clarithromycin susceptibility (126). A retrospective cohort study reported that a triple-drug blister-packaged medication with amoxicillin, clarithromycin, and vonoprazan improved the first-line eradication rate of *H. pylori* in elderly patients (127). Therefore, vonoprazan-based therapy can be a valuable and promising new approach for the treatment of *H. pylori* infection in elderly.

New treatment strategies, including complementary and alternative medicine, are also being considered. Phytomedicines such as traditional Chinese medicine (TCM) treatment can not only improve the eradication rate of *H. pylori*, but also alleviate clinical symptoms and reduce adverse effects (128). *H. pylori* is considered as an “evil qi” in TCM theory. TCM believes that while trying to get rid of the “evil qi,” the “healthy qi” should be strengthened. In other words, it emphasizes on the protection of gastric mucosa and the promotion of immune regulation, which is of great significance in the prevention of *H. pylori*-related diseases. The Fifth Chinese National Consensus Report on the management of *H. pylori* infection suggests that the therapeutic effect of Chinese herbal medicines and Chinese patent medicines on *H. pylori* infection should be studied (11). To date, many studies have been conducted on the efficacy of classical ancient formulas [e.g., Banxia Xiexin decoction (129), Huangqi Jianzhong decoction (130), etc.], empirical prescriptions [Shengjiang Yiyou decoction (131), etc.], and proprietary Chinese medicines [Qingwei Qushi granules (132), Jinghua Weikang capsules (133), etc.] to treat *H. pylori* infection. For example, a randomized, double-blind, placebo-controlled clinical trial showed that the Burdock complex can ameliorate UBT, enhance antioxidant capacity, and reduce inflammatory response in asymptomatic patients with *H. pylori* infection (134). Some Chinese herbal medicines, such as Coptidis Rhizoma (Huang Lian) (135, 136), Scutellariae Radix (Huang Qin) (137), and Polygonum capitatum (Tou Hualiao) (138), also have anti-*H. pylori* effect. The studies have been in-depth and specific to the active components with clear chemical structure, among which berberine, the active component of Coptidis Rhizoma and many other traditional Chinese medicines, has been studied the most. Studies have indicated that berberine exerts antibacterial and anti-inflammatory effects by attenuating the Th17 response triggered by the B cell-activating factor, thus regulating macrophage polarization through the IL-4-STAT6 signaling pathway and suppressing proinflammatory genes and the IRF8-IFN-γ signaling axis (139–141). A meta-analysis has shown that the addition of berberine to standard triple therapy significantly improves *H. pylori* eradication, relieves clinical symptoms, accelerates ulcer healing, and has fewer side effects comparing to the standard triple therapy (142). As per a previous study, the combination of traditional Chinese and Western medicine may overcome the problem of antibiotic resistance, or be useful as a remedy after the failure of antibiotic-based eradication therapy (143). For elderly patients showing contraindications to the use of antibiotics, TCM may be an appropriate option to suppress gastric mucosal inflammation, delay lesion progression, and improve the quality of life.

## Discussions

*H. pylori* infection is considered the most common and controllable risk factor for gastric cancer. Screening and employing appropriate treatment strategies in areas with a high incidence of gastric cancer can effectively reduce its risk, ideally before the occurrence of gastric mucosal atrophy and intestinal metaplasia. Eradication therapy can reduce the risk of gastric cancer and prevent NSAID-induced ulcer in elderly patients. However, the degree of benefit depends on the degree of aging and life expectancy. Due to the decline in physical functions, comorbidities, and the presence of certain combined medications, many older adults have difficulty in following the standard treatment regimen recommended for adults.

Eradication therapy should be used to treat elderly patients only after an adequate risk-benefit assessment. The ideal regimen should be safe and effective, with minimal interaction with other drugs. However, the situation of continuous upgrade of the types and duration of eradication therapy, remains not ideal for the elderly patient. By optimizing the doses, reducing the use of antibiotics, or supplementing complementary drugs, the risk of adverse effects and drug interactions can be effectively reduced. The resurgence of dual therapy, especially the current hot topic, high-dose dual therapy, and vonoprazan, provided new directions. It successfully achieved satisfactory efficacy with fewer medications in normal patients. Further evaluation of its efficacy and safety in elderly patients should be conducted to gather more evidence (e.g., ChiCTR2100045059 in https://www.chictr.org.cn/).

Eradication therapy of *H. pylori* might not benefit all elderly patients. Patients aged >80 years, or those with severe organ function decline, and limited life expectancy, cannot tolerate full dosage and duration of eradication therapy. However, the possible benefits of eradication therapy should not be ignored in case of *H. pylori*-related diseases. Non-antibiotic treatment, typically involving probiotics and natural products that inhibit *H. pylori* activity, might be a complementary and alternative therapy. Several herbal medicines show remarkable anti-*H. pylori* properties (144); however, clinical trials are needed to confirm their effects. In China, integrative medicine is being considered as a new approach for *H. pylori* treatment in older patients, for which, clinical trials are being conducted (e.g., ChiCTR1900028373 in https://www.chictr.org.cn/).

Considering the progress toward an aging society and the high incidence of gastric cancer, more attention needs to be paid to the rational diagnosis and treatment of *H. pylori* infection in the elderly. Elderly patients also need to be finely stratified and treated with appropriate individualized eradication regimens or non-antibiotic regimens.

## Author Contributions

QH and XJ studied the literature and drafted the articles. YC participated in writing. XZ and HY identified the topics, analyzed the literature, presented the outlines, reviewed, and approved the publication of the article. All authors read and approved the final version of the manuscript.

## Funding

This work was supported by the National Natural Science Foundation of China (Grant Number: 81973615) and the Beijing TCM Science and Technology Foundation (Grant Number: JJ2018-105).

## Conflict of Interest

The authors declare that the research was conducted in the absence of any commercial or financial relationships that could be construed as a potential conflict of interest.

## Publisher's Note

All claims expressed in this article are solely those of the authors and do not necessarily represent those of their affiliated organizations, or those of the publisher, the editors and the reviewers. Any product that may be evaluated in this article, or claim that may be made by its manufacturer, is not guaranteed or endorsed by the publisher.
